# New Radiolabeled Exendin Analogues Show Reduced Renal Retention

**DOI:** 10.1021/acs.molpharmaceut.3c00117

**Published:** 2023-06-02

**Authors:** Lieke Joosten, Cathelijne Frielink, Theodorus J. P. Jansen, Daphne Lobeek, Fritz Andreae, Mark Konijnenberg, Sandra Heskamp, Martin Gotthardt, Maarten Brom

**Affiliations:** † Department of Medical Imaging, Nuclear Medicineof Medical Imaging, Radboud University Medical Center, 6525 GA Nijmegen, The Netherlands; ‡ Forschungs- und Entwicklungs GmbH, piCHEM, Parkring 3, 8074 Grambach, Austria

**Keywords:** Ga-68, Lu-177, exendin, renal retention, insulinomas, PET/CT

## Abstract

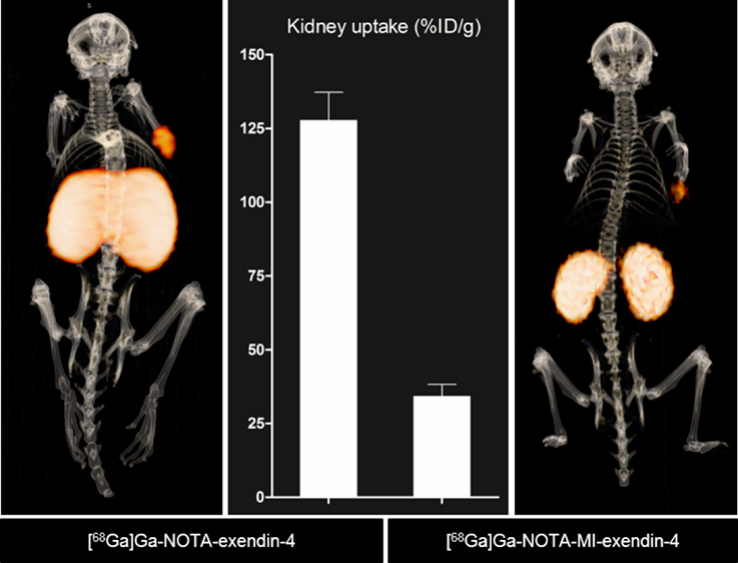

PET imaging of the glucagon-like peptide-1 receptor (GLP-1R) using radiolabeled exendin is a promising imaging method to detect insulinomas. However, high renal accumulation of radiolabeled exendin could hamper the detection of small insulinomas in proximity to the kidneys and limit its use as a radiotherapeutic agent. Here, we report two new exendin analogues for GLP-1R imaging and therapy, designed to reduce renal retention by incorporating a cleavable methionine–isoleucine (Met-Ile) linker. We examined the renal retention and insulinoma targeting properties of these new exendin analogues in a nude mouse model bearing subcutaneous GLP-1R-expressing insulinomas. NOTA or DOTA was conjugated via a methionine–isoleucine linker to the C-terminus of exendin-4 (NOTA-MI-exendin-4 or DOTA-MI-exendin-4). NOTA- and DOTA-exendin-4 without the linker were used as references. The affinity for GLP-1R was determined in a competitive binding assay using GLP-1R transfected cells. Biodistribution of [^68^Ga]Ga-NOTA-exendin-4, [^68^Ga]Ga-NOTA-MI-exendin-4, [^177^Lu]Lu-DOTA-exendin-4, and [^177^Lu]Lu-DOTA-MI-exendin-4 was determined in INS-1 tumor-bearing BALB/c nude mice, and PET/CT was acquired to visualize renal retention and tumor targeting. For all tracers, dosimetric calculations were performed to determine the kidney self-dose. The affinity for GLP-1R was in the low nanomolar range (<11 nM) for all peptides. *In vivo* biodistribution revealed a significantly lower kidney uptake of [^68^Ga]Ga-NOTA-MI-exendin-4 at 4 h post-injection (p.i.) (34.2 ± 4.2 %IA/g), compared with [^68^Ga]Ga-NOTA-exendin-4 (128 ± 10 %IA/g). Accumulation of [^68^Ga]Ga-NOTA-MI-exendin-4 in the tumor was 25.0 ± 8.0 %IA/g 4 h p.i., which was similar to that of [^68^Ga]Ga-NOTA-exendin-4 (24.9 ± 9.3 %IA/g). This resulted in an improved tumor-to-kidney ratio from 0.2 ± 0.0 to 0.8 ± 0.3. PET/CT confirmed the findings in the biodistribution studies. The kidney uptake of [^177^Lu]Lu-DOTA-MI-exendin-4 was 39.4 ± 6.3 %IA/g at 24 h p.i. and 13.0 ± 2.5 %IA/g at 72 h p.i., which were significantly lower than those for [^177^Lu]Lu-DOTA-exendin-4 (99.3 ± 9.2 %IA/g 24 h p.i. and 45.8 ± 3.9 %IA/g 72 h p.i.). The uptake in the tumor was 7.8 ± 1.5 and 11.3 ± 2.0 %IA/g 24 h p.i. for [^177^Lu]Lu-DOTA-MI-exendin-4 and [^177^Lu]Lu-DOTA-exendin-4, respectively, resulting in improved tumor-to-kidney ratios for [^177^Lu]Lu-DOTA-MI-exendin-4. The new exendin analogues with a Met-Ile linker showed 2–3-fold reduced renal retention and improved tumor-to-kidney ratios compared with their reference without the Met-Ile linker. Future studies should demonstrate whether [^68^Ga]Ga-NOTA-MI-exendin-4 results in improved detection of small insulinomas in close proximity to the kidneys with PET/CT. [^177^Lu]Lu-DOTA-MI-exendin-4 might open a window of opportunity for exendin-based radionuclide therapy.

## Introduction

Insulinomas are functioning neuroendocrine tumors, which originate from the beta cells in the pancreatic islets of Langerhans. Although the incidence of insulinomas is very low (0.4%), and only a small percentage (<10%) of insulinomas are malignant, the clinical symptoms, namely, hypoglycemia, are severe and require adequate treatment.^1,2^ Currently, the treatment of choice for insulinomas is surgical removal of the lesion or partial pancreatectomy. Accurate localization of insulinomas to guide therapeutic intervention is essential. Since 10–27% of insulinomas are not detected intraoperatively,^2^ preoperative and/or intraoperative localization of insulinomas is of pivotal importance to identify the tumor lesion and minimize unnecessary surgical intervention. A promising clinical imaging strategy to visualize (benign) insulinomas and native beta cells in the pancreas is based on glucagon-like peptide-1 receptor (GLP-1R) targeting using radiolabeled exendin and SPECT/CT or PET/CT.^3−5^ The GLP-1R is highly and specifically expressed on beta cells. Furthermore, recent studies suggest that this imaging method is preferred over somatostatin receptor scintigraphy and may be considered for the localization of insulinomas instead of somatostatin PET/CT.^4,6^ Exendin-4 is a stable analogue of the natural, unstable GLP-1 ligand, binds with high affinity to the GLP-1R,^7^ and shows high accumulation in insulinomas upon intravenous administration. However, radiolabeled exendin-4 also shows a high renal uptake as a result of tubular reabsorption in the kidneys.^8^

In certain cases, a high renal uptake may be of concern as insulinomas are often very small in size (≤2 cm)^1,2^ and located in close proximity to the kidneys, especially lesions located in the pancreatic tail.^3,4,9,10^ This may hamper visualization of these lesions. Another concern is that [^68^Ga]Ga-exendin PET scans of patients can display a white halo around the kidneys, a serious reconstruction artifact as a result of the high kidney uptake.^11^ These artifacts cannot be solved by using different reconstruction algorithms and hamper the detection of tumor lesions.

High kidney uptake also limits the use of radiolabeled exendin for therapeutic applications. A dosimetry study explored the absorbed kidney dose of [^177^Lu]Lu-DO3A-VS-Cys^40^-exendin-4 by extrapolating rat data to human absorbed doses, showing that the maximum tolerated kidney dose (23 Gy) limits the amount of activity that could be injected (to 4 GBq).^12^ Such a high absorbed kidney dose would allow for only one cycle of treatment of [^177^Lu]Lu-DO3A-VS-Cys^40^-exendin-4. This is in contrast with a dose–response study using [^177^Lu]Lu-DOTATATE for the treatment of pancreatic neuroendocrine tumors, where two to six therapeutic cycles of 7.4 GBq are feasible.^13^ Hence, reducing the exendin kidney uptake may be beneficial for diagnostic purposes, but it is essential before considering radiolabeled exendin for therapy.

Several successful strategies to reduce the renal uptake of radiolabeled peptides have been described. For example, van Eerd et al. were the first to show a reduced renal uptake of ^111^In-labeled octreotide by pre-injection of Gelofusine.^14^ Likewise, Vegt et al. obtained the same results in human volunteers.^15^ In a rat study, Gelofusine was shown to reduce the renal uptake of ^111^In-labeled exendin by almost 19%.^16^ A similar effect was found in humans.^17^ Furthermore, Hammond et al. used the amino acids lysine and arginine to block the tubular re-uptake of [^111^In]In-DTPA-octreotide,^18^ which was also shown to be effective in patients with neuroendocrine tumors.^19^ Blocking the tubular reabsorption of radiolabeled exendin using arginine and lysine is, however, not effective.^16^ Thus, in order to further reduce the kidney uptake of radiolabeled exendin, a new approach is demanded.

Wu et al. have shown that a methionine conjugate, labeled with gallium-67, was rapidly excreted from the kidneys.^20^ Based on the conclusions of the latter study, Uehara et al. reported a methionine–isoleucine linker between NOTA and a Fab fragment of a mAb against HER2. They observed a reduction in kidney uptake of almost 88%, which could be attributed to the additional linker that was introduced.^21^ These two studies evoked the question whether the translation of the methionine–isoleucine linker to exendin-4 could help to reduce the renal uptake of exendin. Based on literature, the addition of a cleavable linker seems to be more effective in reducing the renal uptake of radiolabeled exendin than co-administration of Gelofusine or lysine, which only resulted in a 19% decrease and even a 6% increase in renal uptake, respectively.^16,17^ Furthermore, although very rare, side effects or allergic reactions to these agents can be avoided when using a cleavable peptide to reduce kidney uptake.

The purpose of this study was to develop and characterize two novel exendin analogues with reduced renal retention suitable for GLP-1R imaging and therapy. We have selected the radionuclides gallium-68 (^68^Ga) and lutetium-177 (^177^Lu), because they are used for insulinoma imaging and radionuclide therapy, respectively. The analogues were based on exendin-4, with a methionine–isoleucine linker incorporated between the chelator and the peptide. The renal retention and insulinoma targeting properties of these new exendin analogues labeled with ^68^Ga or ^177^Lu have been evaluated in a xenograft mouse model bearing subcutaneous GLP-1R-expressing insulinomas. Furthermore, this concept was validated using an additional chelator (NODAGA).

## Materials and Methods

### Peptides and Radionuclides

NOTA-exendin-4, **Lys**^40^[Mep((S-)NOTA-**M**et-**I**le-Mal-)]exendin-4 (referred to as NOTA-MI-exendin-4), DOTA-exendin-4, **Lys**^40^[Mep((S-)DOTA-**M**et-**I**le-Mal-)]exendin-4 (referred to as DOTA-MI-exendin-4), NODAGA-exendin-4, **Lys**^40^[Mep((S-)NODAGA-**M**et-**I**le-Mal-)]exendin-4 (referred to as NODAGA-MI-exendin-4), and [Lys^40^(DTPA)]exendin-3 were purchased from piCHEM (Graz, Austria).

Control peptides [Lys^40^(DTPA)]exendin-3, [Lys^40^(NOTA)]exendin-4, [Lys^40^(DOTA)]exendin-4, and [Lys^40^(NODAGA)]exendin-4 were conjugated with p-NCS-benzyl-functionalized DTPA, NOTA, DOTA, or NODAGA to the ε-amino group of the lysine (L-form) at position 40.

**Figure 1:**
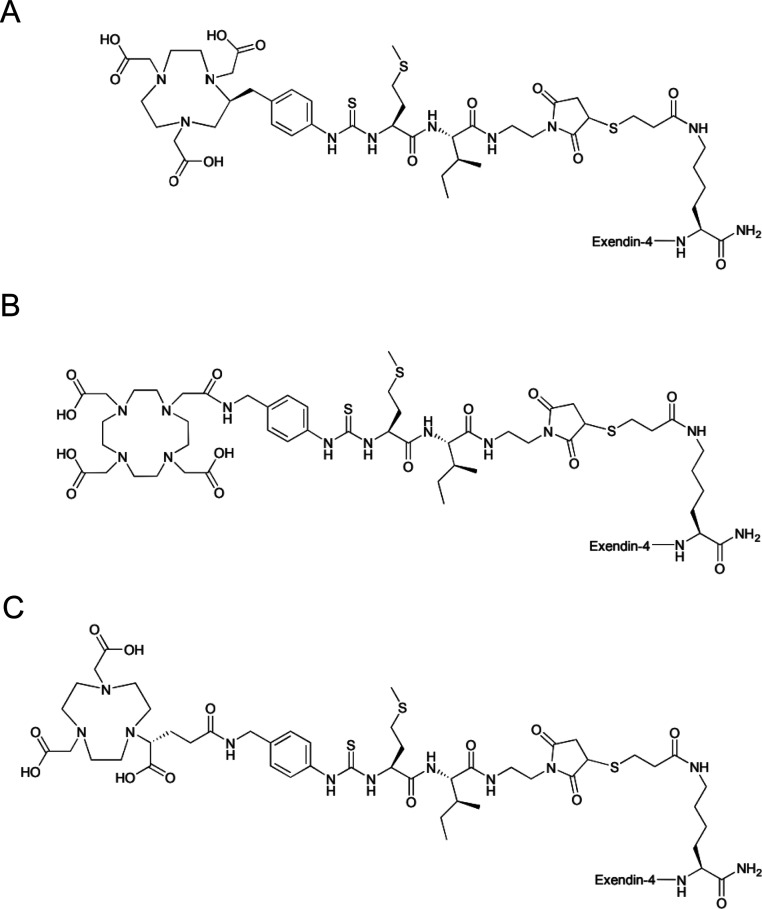
Structures of NOTA-MI-exendin-4 (A), DOTA-MI-exendin-4 (B), and NODAGA-MI-exendin-4 (C).

^111^InCl_3_ was obtained from Curium (Petten, The Netherlands), and ^68^Ga was eluted from a ^68^Ge/^68^Ga generator (GalliaPharm Generator, Eckert & Ziegler Eurotope, Berlin, Germany) with 0.1 M HCl (Rotem GmbH, Israel) at a flow rate of 1 mL/min. No carrier added ^177^LuCl_3_ was obtained from ITM (Isotope Technologies Munich, Germany).

To obtain NOTA-MI-exendin-4, DOTA-MI-exendin-4, and NODAGA-MI-exendin-4, exendin-4 was modified with a Mep (mercapto-propionic acid) at the lysine side chain at position 40. The spacer molecule, methionyl-isoleucine (both the L-form), was modified at the C-terminus with N-(2-aminoethyl)maleimide as the thiol-reactive cross-linker, whereas the corresponding chelator (enantiomerically pure (*S*)-NOTA-Bn-SCN, DOTA-Bn-SCN, or enantiomerically pure (*R*)-NODAGA-Bn-SCN) was attached at the N-terminus of methionine. Finally, the chelator-spacer group was conjugated via the maleimide to the mercapto-propionyl moiety at the lysine of exendin-4 at position 40. An overview of the structure of these peptides is given in Figure 1 and Table S1.

### Radiolabeling of Exendin-4 Analogues with ^68^Ga

The NOTA- and NODAGA-conjugated peptides were labeled with ^68^Ga by adding 2.5 M HEPES (4-(2-hydroxyethyl)-1-piperazineethanesulfonic acid, Sigma Aldrich) to 35–100 MBq ^68^Ga in ultrapure 0.1 M HCl (HEPES:^68^Ga = 1:12) and 1 μg of the peptide. After incubation at 95 °C for 5 min, ethylenediaminetetraacetic acid (EDTA) (Sigma Aldrich) and 10% Tween-80 were added to a final concentration of 5 mM and 0.1%, respectively. Quality control was performed by reversed-phase high-performance liquid chromatography (RP-HPLC) and instant thin-layer chromatography (ITLC). RP-HPLC was performed using a C_18_ reversed-phase column (Alltima; 4.6 mm × 25 cm; Grace, Breda, The Netherlands). For elution, a linear gradient of 0.1% trifluoroacetic acid (Lab-Scan, Analytical Sciences, Brussels, Belgium) in acetonitrile (3–100% over 10 min) with a flow rate of 1 mL/min was used. The ^68^Ga-colloid content was determined by ITLC: with 1.25 M NH_4_OAc, pH 5.5: dimethylformamide (1:1) as a mobile phase and silica-gel strips (ITLC-SG Biodex, Shirley, NY, USA) as a solid phase (R_f_^68^Ga-hydroxide = 0, R_f_^68^Ga-labeled peptide and ^68^Ga-EDTA = 1).^22^ Purification was performed by solid-phase extraction using an HLB (hydrophilic–lipophilic balance reversed-phase sorbent) cartridge (Waters Oasis, Milford, MA, USA). Activation and washing of the cartridge were performed with 1 mL of 100% ethanol and 3 mL of water, respectively. Serum stability was determined as described in the Supporting Information.

### Radiolabeling of Exendin-4 Analogues with ^177^Lu

DOTA-exendin-4 and DOTA-MI-exendin-4 were labeled with ^177^Lu by incubating 100 μL of metal-free 0.5 M MES [2-(*N*-morpholino)ethanesulfonic acid, Sigma Aldrich, St. Louis, MO, USA], pH 5.5, 15 MBq ^177^Lu, and 1 μg of the respective peptide for 25 min at 95 °C. Subsequently, ∼12 μL of 10% Tween-80 and ∼1.2 μL of 50 mM EDTA were added to a final concentration of 0.1% and 5 mM, respectively. Purification was performed by solid-phase extraction and analysis of the labeling efficiency was carried out by RP-HPLC as described above. Serum stability was analyzed as described in the Supporting Information.

### Radiolabeling of DTPA-Exendin-3 with ^111^In

DTPA-exendin-3 was labeled with ^111^In by mixing 150 MBq of ^111^InCl_3_ (240 μL) with metal-free 0.5 M MES [2-(*N*-morpholino)ethanesulfonic acid (1200 μL), Sigma Aldrich, St. Louis, MO, USA], pH 5.5, and 1 μg of the peptide, followed by incubation at room temperature for 20 min. After labeling, 10% Tween-80 (16 μL) and 50 mM EDTA (160 μL) were added to a final concentration of 0.1% and 5 mM, respectively. Purification by solid-phase extraction and analysis of the labeling efficiency by RP-HPLC were carried out as described above.

### Cell Culture

Chinese hamster lung (CHL) cells transfected with the human GLP-1R (a kind gift of Brigitte Lankat-Buttgereit, Marburg^23^) were maintained in Dulbecco’s modified Eagle’s medium (DMEM) GlutaMAX (Gibco, Invitrogen, Breda, The Netherlands), supplemented with 10% fetal calf serum, 100 units/mL penicillin, Geneticin (G418) sulfate solution (0.5 mg/mL final concentration), 100 μg/mL streptomycin, 1 mM sodium pyruvate, and 0.1 mM non-essential amino acids. The INS-1 cell line (rat insulinoma) was maintained in the RPMI-1640 medium supplemented with 10% fetal bovine serum, 2 mM glutamine, 10 mM HEPES, 50 μM β-mercaptoethanol, 1 mM sodium pyruvate, 100 units/mL penicillin, and 100 μg/mL streptomycin.^24^ Cells were maintained in a humidified 5% CO_2_ atmosphere at 37 °C.

### Competitive Binding Assay (IC_50_)

A competitive binding assay using CHL-GLP-1R cells was performed to compare the affinity of the MI-peptides with their respective control peptides. Cells were grown to confluency overnight at 37 °C, after plating 1 × 10^6^ cells per well in a 6-well plate. Cells were washed with 1 mL of binding buffer (DMEM GlutaMAX + 0.5% bovine serum albumin), and a trace amount of ^111^In-labeled DTPA-exendin-3 (730 GBq/μmol, labeling was performed as previously described) (0.8 kBq, 0.9 fmol) was added to the cells, together with increasing amounts of unlabeled NOTA-exendin-4, NOTA-MI-exendin-4, DOTA-exendin-4, DOTA-MI-exendin-4, NODAGA-exendin-4, or NODAGA-MI-exendin-4, ranging from 0.1 to 1000 nM. After incubation for 4 h at 0 °C, cells were washed twice with 1 mL PBS, harvested with 1 mL of 0.1 M NaOH, and the cell-associated activity was measured in a gamma counter (WIZARD, 2480 Automatic Gamma Counter, Perkin Elmer, Boston, MA, USA). The IC_50_ value (half-maximal inhibitory concentration) was calculated using GraphPad Prism using one-site competition (version 5.03, GraphPad Software, San Diego California USA).

### Animal Studies

The project (2015-0071) was approved by the Nijmegen Medical Center animal ethics committee (RUDEC) and the Dutch animal ethics committee (CCD) of the Radboud University and performed according to the Institute of Laboratory Animal Research Guidelines. C3H mice were purchased from Charles River Laboratories (L’Arbresle, France) and BALB/cRJ nu mice from Janvier (Saint-Berthevin, France). Mice were housed in a pathogen-free environment in Mouse IVC Blueline cages (5 mice per cage), had ad libitum access to water and chow, and were allowed to adapt to laboratory conditions for at least 1 week before the start of the experiments.

### Biodistribution and PET/CT of [^68^Ga]Ga-NOTA-Exendin-4 and [^68^Ga]Ga-NOTA-MI-Exendin-4 in Mice with a Subcutaneous INS-1 Tumor

To assess the effect of the Met-Ile linker on the uptake of radiolabeled exendin-4 in tumor tissue, an experiment was conducted in male BALB/cRJ nu mice (13 weeks old) with subcutaneous INS-1 tumors. Mice were inoculated subcutaneously with 3 × 10^6^ INS-1 cells in 200 μL RPMI medium. After growth of the tumor to an appropriate size (approx. 0.1 cm^3^), mice were assigned randomly into groups of five animals. Mice were anesthetized by isoflurane/O_2_ and underwent PET/CT 1, 2, and 4 h after injection with 200 μL of either [^68^Ga]Ga-NOTA-MI-exendin-4 or [^68^Ga]Ga-NOTA-exendin-4 (1.6 MBq, 20 pmol). Two additional groups of mice received a co-injection of the tracer with an excess of the unlabeled peptide (*n* = 2/group). PET/CT images were acquired for 30–40 min with a small-animal PET/CT scanner (Inveon; Preclinical Solutions, Siemens Medical Solutions USA, Inc., Knoxville, TN, USA). Reconstruction of the images was performed as follows: OSEM3D/SPMAP reconstruction, 256 × 256 matrix, 2 OSEM3D iterations, 18 MAP iterations, and a resolution of 0.075 mm uniform variance. The settings for the CT were as follows: a spatial resolution of 113 μm, 80 kV, 500 μA, and an exposure time of 300 ms. After PET/CT acquisition, mice were euthanized by CO_2_ asphyxiation, and the biodistribution of the radiolabeled peptides was determined by weighing the dissected organs and measuring them in a gamma counter. For each tissue sample, the uptake was calculated and presented as the percentage of the injected activity per gram tissue (%IA/g).

### Biodistribution of [^177^Lu]Lu-DOTA-Exendin-4 and [^177^Lu]Lu-DOTA-MI-Exendin-4 in Mice with a Subcutaneous INS-1 Tumor

Female BALB/cRJ nu mice (*n* = 5/group) with a subcutaneous INS-1 tumor (3 × 10^6^ INS-1 cells in 200 μL RPMI medium) were randomly assigned into groups of five and intravenously injected with 200 μL of 1.5 MBq (20 pmol) [^177^Lu]Lu-DOTA-exendin-4 or [^177^Lu]Lu-DOTA-MI-exendin-4. After 15 and 30 min and 4, 24, 48, and 72 h mice were euthanized, and the biodistribution of the compounds was performed as described above. Two groups of mice (*n* = 2/group) were co-injected with an excess of the unlabeled peptide, and the biodistribution was assessed 4 h after injection.

### Biodistribution of [^68^Ga]Ga-NOTA-Exendin-4, [^68^Ga]Ga-NOTA-MI-Exendin-4, [^68^Ga]Ga-NODAGA-Exendin-4, and [^68^Ga]Ga-NODAGA-MI-Exendin-4 in Healthy Mice

We have evaluated the combination of the methionine–isoleucine linker with Ga-68-labeled NODAGA-exendin-4 in female C3H mice 8–13 weeks old. Mice received an intravenous injection with 200 μL of 20 pmol of either [^68^Ga]Ga-NOTA-MI-exendin-4 (1.7 MBq), [^68^Ga]Ga-NOTA-exendin-4 (1.4 MBq), [^68^Ga]Ga-NODAGA-MI-exendin-4 (4.1 MBq), or [^68^Ga]Ga-NODAGA-exendin-4 (3.7 MBq) (*n* = 5 per group). At 1, 2, and 4 h after injection, the biodistribution was determined as described above. Additional groups of mice (*n* = 2) received an excess of the unlabeled (2 nmol) peptide (the same peptide as the tracer) to determine receptor-mediated binding, and their biodistribution was determined 4 h after injection.

### Dosimetry

Biodistribution data of [^68^Ga]Ga-NOTA-exendin-4, [^68^Ga]Ga-NOTA-MI-exendin-4, [^177^Lu]Lu-DOTA-exendin-4, and [^177^Lu]Lu-DOTA-MI-exendin-4 in mice with a subcutaneous INS-1 tumor were used for dosimetric calculations. The kidney self-dose was calculated based on the activity distribution of the tracer in the kidneys. Time-integrated activity coefficients (MBq-h/MBq) were calculated using mono-exponential curve-fitting between the timepoints. The last timepoint for the time-integrated activity coefficients was determined using 10 times the radioactive half-life of ^68^Ga for NOTA-exendin-4 and 10 times the biological half-life of the peptide for NOTA-MI-exendin-4. OLINDA/EXM software (version 2.1) was used to calculate the absorbed kidney dose in mice using the 25 g RADAR model.

The mice data were then used to extrapolate the expected absorbed kidney doses per injected activity to humans using the equation:^25,26^
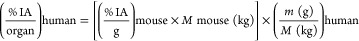
with  the percentage of the injected activity concentration,  the percentage of the injected activity per organ, *m* the organ mass, and *M* the total human or mouse body weight. Subsequently, the extrapolated human time-integrated activity coefficients were used as input in an 80 kg male reference model in OLINDA/EXM software.

### Statistical Analysis

All data were analyzed using GraphPad Prism software version 5.03 for Windows. Student’s *t*-test or one-way ANOVA followed by Tukey’s test were used to determine significance. For the IC_50_ binding assay, the *F*-test was used to manually calculate significance. A *p*-value below 0.05 was considered significant. All data are presented as mean ± standard deviation.

## Results

### Radiolabeling, IC_50_, and Serum Stability

All in vitro results are summarized in Table 1, Figures S1 and S2 and include molar activity, radiochemical purity after purification, 50% inhibitory concentration, and serum stability for all exendin-4 analogues.

**Table 1:** Molar Activity, Radiochemical Purity, IC_50_ Values and 95% Confidence Intervals (in nM), and Serum Stability of Exendin-4 Analogues

peptide	molar activity (GBq/μmol)	radiochemical purity after purification (%)	IC_50_ (nM)	serum stability
NOTA-exendin-4	167 (^68^Ga)	>98	6.3 (95% CI: 4.6–8.6)	>90% after 2 h
			4.0^a^ (95% CI: 2.5–6.3)	
NOTA-MI-exendin-4	183 (^68^Ga)	>98	9.5^a^ (95% CI: 7.0–12.8)	>90% after 2 h
NODAGA-exendin-4	467 (^68^Ga)	>95	4.7 (95% CI: 3.4–6.4)	>90% after 2 h
NODAGA-MI-exendin-4	531 (^68^Ga)	>85	5.2 (95% CI: 3.8–7.2)	>45% after 2 h
DOTA-exendin-4	84 (^177^Lu)	>95	8.7 (95% CI: 6.5–16.5)	>65% after 24 h
DOTA-MI-exendin-4	87 (^177^Lu)	>90	10.4 (95% CI: 7.3–10.3)	>80% after 24 h

a These are from a different experiment. CI: 95% confidence interval.

### Biodistribution and PET/CT of [^68^Ga]Ga-NOTA-Exendin-4 and [^68^Ga]Ga-NOTA-MI-Exendin-4 in Mice with a Subcutaneous INS-1 Tumor

**Figure 2:**
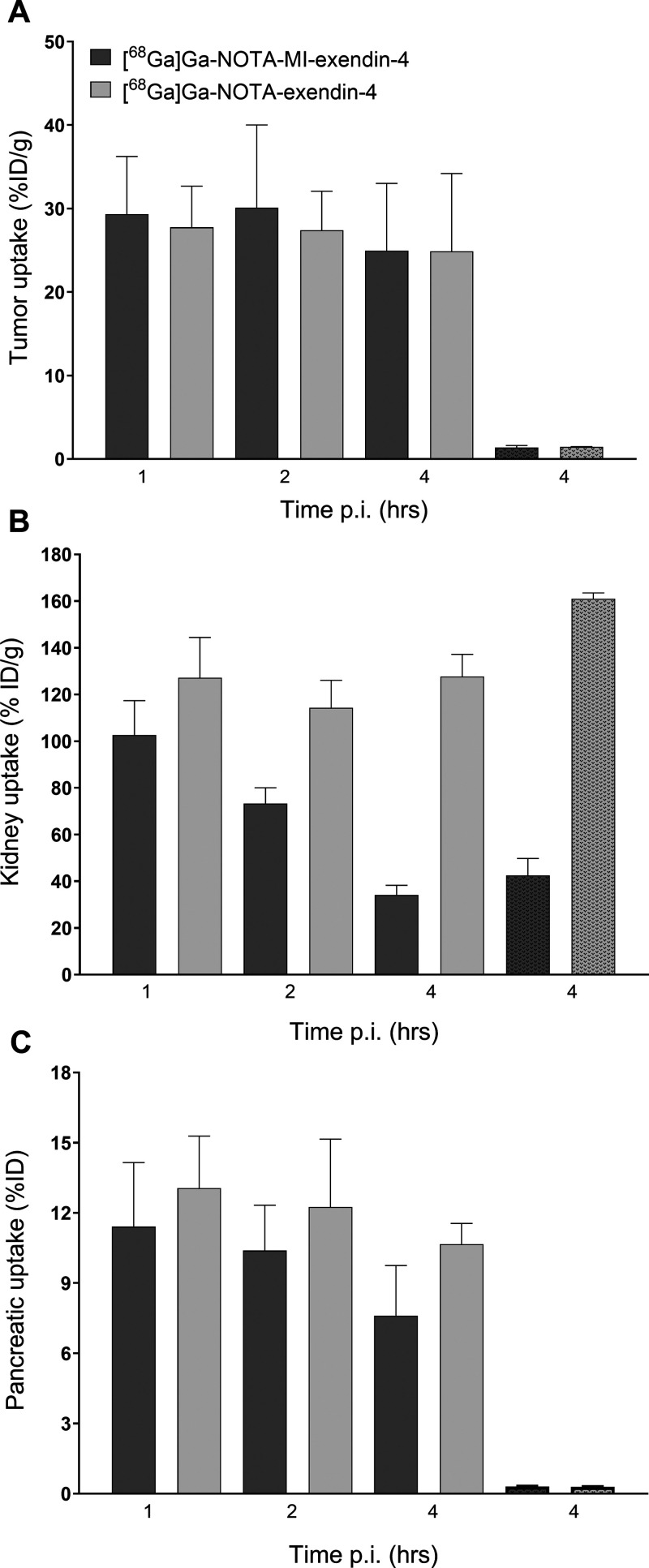
Biodistribution of ^68^Ga-labeled NOTA-exendin-4 and NOTA-MI-exendin-4 in BALB/c nude mice bearing subcutaneous CHL-GLP-1R tumors 1, 2, and 4 h after injection. Tumor uptake (A), kidney uptake (B), and pancreatic uptake (C). Receptor saturation was performed by co-injection of a 100-fold excess of the unlabeled peptide 4 h after injection (dotted bars). Values are expressed as the percentage injected activity per gram of tissue (%IA/g) (*n* = 4–5 mice per group, error bars SD).

Figure 2A shows that the high accumulation of both [^68^Ga]Ga-NOTA-exendin-4 and [^68^Ga]Ga-NOTA-MI-exendin-4 in INS-1 tumors was stable over time ([^68^Ga]Ga-NOTA-MI-exendin-4: 29.3 ± 6.9 %IA/g at 1 h and 25.0 ± 8.0 %IA/g at 4 h post-injection (p.i.); [^68^Ga]Ga-NOTA-exendin-4: 27.8 ± 4.9 %IA/g at 1 h and 24.9 ± 9.3 %IA/g at 4 h p.i.). Furthermore, a remarkably reduced kidney uptake of 66% for [^68^Ga]Ga-NOTA-MI-exendin-4 was seen over 4 h (Figure 2B and Table 3), whereas there was no reduction for [^68^Ga]Ga-NOTA-exendin-4. The tumor-to-kidney ratio was 0.19 ± 0.06 for [^68^Ga]Ga-NOTA-exendin-4, compared to 0.75 ± 0.30 for [^68^Ga]Ga-NOTA-MI-exendin-4 (*p* < 0.001) and was most optimal 4 h after injection (Table 2). The complete data of this biodistribution study are shown in Table S2. These results are in line with data from biodistribution studies in healthy female CH3 mice with [^68^Ga]Ga-NOTA-exendin-4, [^68^Ga]Ga-NOTA-MI-exendin-4, [^68^Ga]Ga-NODAGA-exendin-4, and [^68^Ga]Ga-NODAGA-MI-exendin-4 (Supporting Information, Table S3). Since the NOTA-conjugated compounds had a much more favorable kidney uptake than the NODAGA-conjugated compounds, these were further examined in an imaging study with tumor-bearing mice.

**Table 2:** Tumor-to-Normal Organ Ratios and Pancreas-to-Kidney Ratio for [^68^Ga]Ga-NOTA-Exendin-4 and [^68^Ga]Ga-NOTA-MI-Exendin-4^a^

time p.i. (h)	tumor-to-blood	tumor-to-pancreas	tumor-to-kidney	pancreas-to-kidney
[^68^Ga]Ga-NOTA-Exendin-4				
1	111 ± 19	2.22 ± 0.80	0.22 ± 0.03	0.10 ± 0.02
2	197 ± 35	2.43 ± 1.07	0.24 ± 0.05	0.11 ± 0.02
4	226 ± 66	2.29 ± 0.72	0.19 ± 0.06	0.08 ± 0.01
[^68^Ga]Ga-NOTA-MI-Exendin-4				
1	90 ± 23	2.69 ± 0.88	0.29 ± 0.05	0.11 ± 0.02
2	135 ± 45	3.05 ± 1.26	0.42 ± 0.15	0.14 ± 0.02
4	189 ± 74	3.67 ± 2.28	0.75 ± 0.30	0.22 ± 0.07

a Mean ± SD are shown.

**Figure 3:**
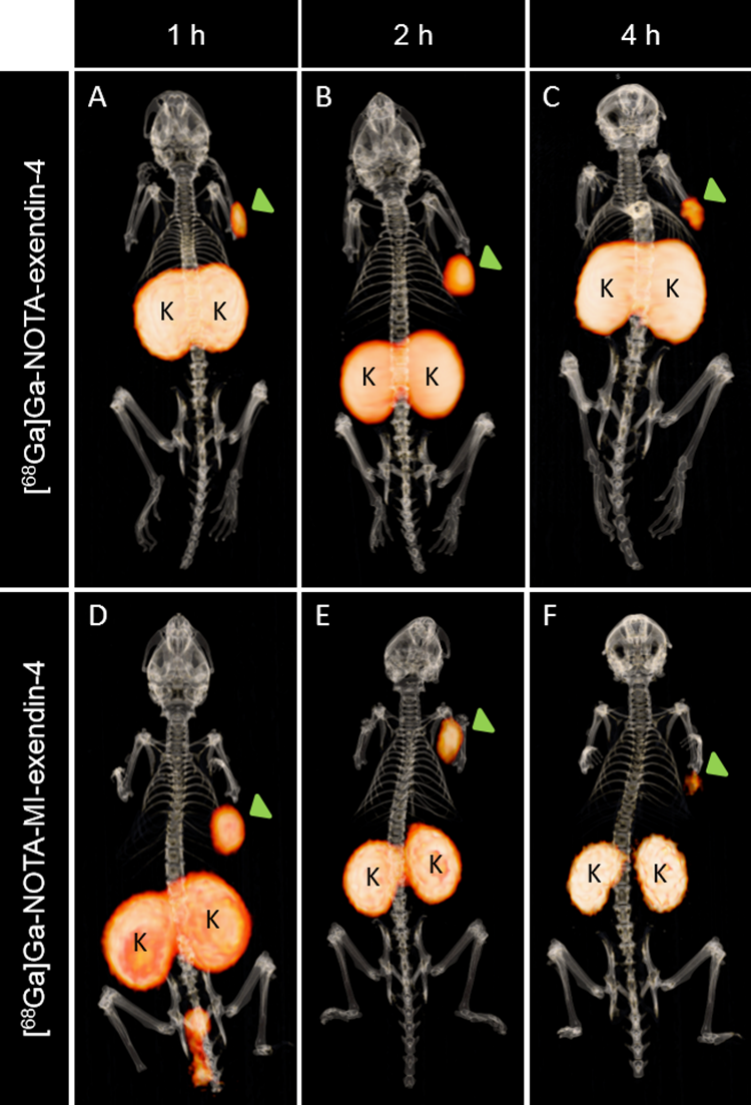
Fused PET/CT images of BALB/c nude mice bearing subcutaneous CHL-GLP-1R tumors (green arrow). Mice were injected with 1.6 MBq of either [^68^Ga]Ga-NOTA-exendin-4 (A–C) or [^68^Ga]Ga-NOTA-MI-exendin-4 (D–F). Images were obtained 1 h (A,D), 2 h (B,E), and 4 h (C,F) after injection. Kidneys are indicated with K.

The images in Figure 3F reveal reduced renal uptake and improved discrimination between the left and right kidney when using ^68^Ga-labeled exendin-4 with the Met-Ile linker, while maintaining a significant signal in the INS-1 tumor. This is in contrast to the images of [^68^Ga]Ga-NOTA-exendin-4, where the kidneys could not be discriminated from each other due to the high renal retention (Figure 3C).

### Biodistribution of [^177^Lu]Lu-DOTA-Exendin-4 and [^177^Lu]Lu-DOTA-MI-Exendin-4 in Mice with a Subcutaneous INS-1 Tumor

**Figure 4:**
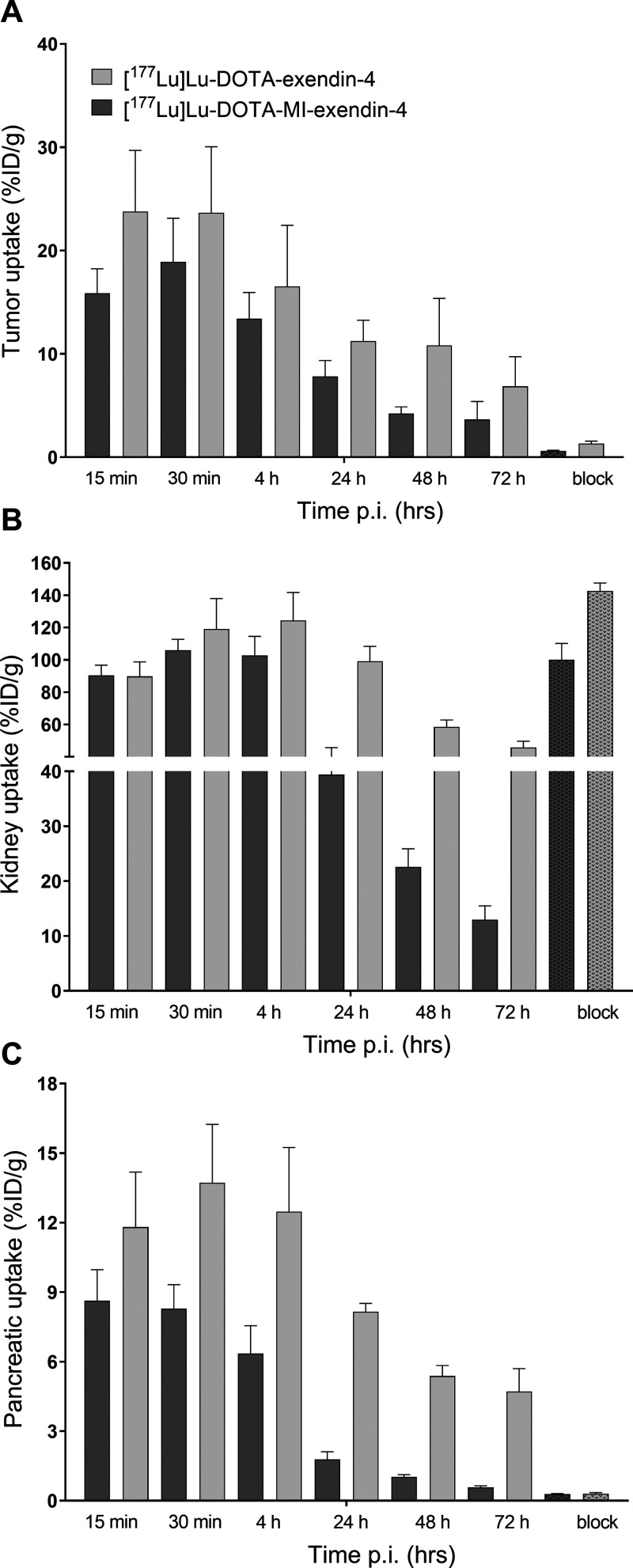
Biodistribution of ^177^Lu-labeled DOTA-exendin-4 and DOTA-MI-exendin-4 in BALB/c nude mice bearing subcutaneous CHL-GLP-1R tumors 15 and 30 min and 4, 24, 48, and 72 h after injection. Tumor uptake (A), kidney uptake (B), and pancreatic uptake (C). Receptor saturation was performed by co-injection of a 100-fold excess of the unlabeled peptide 4 h after injection (dotted bars). Values are expressed as the percentage injected activity per gram of tissue (%IA/g) (*n* = 4–5 mice per group, error bars SD).

As depicted in Figure 4A, the biodistribution of ^177^Lu-labeled DOTA-exendin-4 and DOTA-MI-exendin-4 revealed that for both compounds the tumor uptake declines over time, although this reduction was not significant from one timepoint to the next. Furthermore, [^177^Lu]Lu-DOTA-exendin-4 seems to have a higher tumor uptake than [^177^Lu]Lu-DOTA-MI-exendin-4, but this difference was never significant. The pancreatic uptake was relatively stable for the first 4 h, after which a remarkable drop in the uptake was observed [12.5 ± 2.8 %IA/g to 4.7 ± 1.0 %IA/g for [^177^Lu]Lu-DOTA-exendin-4 (*p* < 0.001) and 6.4 ± 1.2 %IA/g to 0.6 ± 0.06 %IA/g for [^177^Lu]Lu-DOTA-MI-exendin-4 (*p* < 0.001)]. This decline continued for both compounds and was much more pronounced for [^177^Lu]Lu-DOTA-MI-exendin-4, where the uptake 72 h p.i. was similar to the blocked conditions (Figure 4C). The kidney uptake was similar for both compounds for the first 4 h and relatively stable. Between 4 and 24 h, a reduction of 62% for [^177^Lu]Lu-DOTA-MI-exendin-4 was observed, and only 20% for [^177^Lu]Lu-DOTA-exendin-4 (Table 3). Moreover, after 72 h, the kidney uptake of [^177^Lu]Lu-DOTA-MI-exendin-4 was only 13.0 ± 2.5 %IA/g, whereas it was 45.8 ± 3.9 %IA/g for [^177^Lu]Lu-DOTA-exendin-4 (Figure 4B). In Table 4, the tumor-to-normal organ ratios are given. The complete data of this biodistribution are shown in Table S4.

**Table 3:** Reduction of Kidney Uptake per Peptide between the Different Timepoints^a^

	[^68^Ga]Ga-NOTA-exendin-4	[^68^Ga]Ga-NOTA-MI-exendin-4
1–2 h	10.1%	28.5%
2–4 h	–11.7%	53.5%
1–4 h	–0.4%	66.7%

	[^177^Lu]Lu-DOTA-exendin-4	[^177^Lu]Lu-DOTA-MI-exendin-4
15–30 min	–32.7%	–17.3%
30 min to 4 h	–4.6%	3.1%
4–24 h	20.3%	61.7%
24–48 h	41.0%	42.7%
48–72 h	21.8%	42.5%
15 min to 72 h	49.0%	85.7%

a Note that a negative value thus indicates an increase in kidney uptake.

**Table 4:** Tumor-to-Normal Organ Ratios and Pancreas-to-Kidney Ratio for [^177^Lu]Lu-DOTA-Exendin-4 and [^177^Lu]Lu-DOTA-MI-Exendin-4^a^

time p.i.	tumor-to-blood	tumor-to-pancreas	tumor-to-kidney	pancreas-to-kidney
[^177^Lu]Lu-DOTA-Exendin-4				
15 min	9.70 ± 1.92	2.01 ± 0.25	0.27 ± 0.06	0.13 ± 0.02
30 min	28 ± 6	1.71 ± 0.24	0.20 ± 0.04	0.12 ± 0.02
4 h	163 ± 51	1.35 ± 0.42	0.15 ± 0.02	0.10 ± 0.01
24 h	381 ± 90	1.38 ± 0.22	0.11 ± 0.03	0.08 ± 0.01
48 h	812 ± 390	2.02 ± 0.85	0.18 ± 0.07	0.09 ± 0.01
72 h	860 ± 326	1.52 ± 0.72	0.15 ± 0.06	0.10 ± 0.02
[^177^Lu]Lu-DOTA-MI-Exendin-4				
15 min	7.98 ± 1.57	1.88 ± 0.40	0.18 ± 0.03	0.10 ± 0.01
30 min	19 ± 6	2.31 ± 0.59	0.18 ± 0.03	0.08 ± 0.01
4 h	79 ± 19	2.16 ± 0.49	0.13 ± 0.03	0.06 ± 0.01
24 h	231 ± 63	4.41 ± 0.83	0.20 ± 0.04	0.05 ± 0.01
48 h	320 ± 52	4.11 ± 0.44	0.19 ± 0.01	0.05 ± 0.01
72 h	521 ± 174	6.11 ± 2.34	0.28 ± 0.09	0.05 ± 0.01

a Mean ± SD are shown.

### Dosimetry

The estimated absorbed kidney doses per injected activity for mice were 266 and 232 mSv/MBq for [^68^Ga]Ga-NOTA-exendin-4 and [^68^Ga]Ga-NOTA-MI-exendin-4, respectively. For humans, the estimated absorbed kidney doses were 0.20 and 0.17 mSv/MBq, respectively. The use of [^68^Ga]Ga-NOTA-MI-exendin-4 reduced the estimated absorbed kidney dose in both mice and humans by 12%. For the ^177^Lu-labeled compounds, the reduced absorbed dose was 57%. The estimated absorbed kidney doses per injected activity in mice were 4548 and 1974 mSv/MBq for DOTA-exendin-4 and DOTA-MI-exendin-4, respectively, and for humans 2.05 and 0.89 mSv/MBq, respectively.

## Discussion

Complete surgical removal of insulinomas is the only curative treatment option but remains challenging as insulinomas are often small in size and difficult to localize. Pre-operative GLP-1R imaging with radiolabeled exendin can be used to accurately localize insulinoma lesions; however, some tumors may be missed because they are masked by the high kidney uptake. Furthermore, in rare cases with metastatic GLP-1R positive insulinoma,^27^ which cannot be resected, peptide receptor radionuclide therapy (PRRT) using exendin may be exploited (also for reducing insulin production and symptom control). Here, we demonstrate that novel exendin-4 analogues with a methionine–isoleucine linker show a reduced renal uptake while maintaining high accumulation in subcutaneous GLP-1R-expressing INS-1 tumors. Therefore, our new compounds may improve visualization of small insulinomas located in the vicinity of the kidneys and open a window for PRRT for patients that are not eligible for surgery (i.e., metastasized insulinomas or insulinomas near vessels or ducts).

Tubular reabsorption has been described as one of the major causes of kidney retention of radiolabeled exendin,^16^ but expression of the GLP-1R on porcine proximal tubular cells has been reported as well.^28^ Exendin uptake in the kidneys cannot be blocked by an excess of unlabeled exendin; thus, the contribution of the GLP-1R-mediated uptake in the tubules seems to play only a minor role. In general, peptides enter the kidneys via the bloodstream and are filtered through the glomeruli. Instead of being excreted, the peptides are reabsorbed in the proximal tubules,^29^ most probably via the endocytic receptors megalin and cubilin that are located near the brush border membrane.^8^ After endocytosis, the peptides are cleaved in lysosomes to amino acids by enzymatic degradation.^29^ As the radionuclide–chelator complex is a residualizing compound (“metabolic trapping”), it will be trapped in the lysosomes instead, leading to high accumulation of the tracer.

In this study, [^68^Ga]Ga-NOTA-MI-exendin-4 showed rapid clearance from the kidneys (a reduction of 66% between 1 and 4 h) and therefore had a much shorter renal retention time than [^68^Ga]Ga-NOTA-exendin-4. This is in line with the findings reported by Uehara et al. A reduction in kidney uptake was demonstrated after introduction of a methionine–isoleucine linker between the chelator and the compound.^21^ Later, they published an antibody fragment with a newly designed cleavable linker, methionine–valine–lysine (MVK), which revealed a further reduced renal uptake in comparison to the methionine–isoleucine linker. More recently, an exendin-4 analogue containing the MVK linker was developed. Similar to our study, they reported a stable tumor uptake (around 25 %IA/g at 1 and 2 h p.i.) of the ^68^Ga-labeled compound and a striking decrease in renal accumulation. Two hours p.i., the uptake of [^68^Ga]Ga-NOTA-MVK-Cys^40^-Leu^14^-exendin-4 was 33% of the control. Similarly, the kidney uptake of our ligand was 36% of the control, which was even more pronounced 4 h after injection (73% of the control).^30,31^

Importantly, while [^68^Ga]Ga-NOTA-MI-exendin-4 showed a reduced uptake in kidneys and other GLP-1R organs (pancreas, lungs, and duodenum) already 1 h after injection, the uptake in the INS-1 tumor was unaltered. A stable tumor uptake combined with a reduced pancreatic uptake is an advantage, as this improves the tumor-to-pancreas ratio and eventually could improve the detection of insulinomas, as almost all insulinomas are located in the pancreas. In addition, the pancreas-to-kidney and tumor-to-kidney ratio of [^68^Ga]Ga-NOTA-MI-exendin-4 were almost three and four times higher (respectively) than those for [^68^Ga]Ga-NOTA-exendin-4 4 h after injection. We have previously shown that even 4 h after injection of 100 MBq of ^68^Ga-labeled exendin-4, PET scans can be acquired of patients with an insulinoma with a very good image quality.

Although we found a major reduced renal uptake for [^68^Ga]Ga-NOTA-MI-exendin-4 within the first 4 h, for [^177^Lu]Lu-DOTA-MI-exendin-4, the effect of the cleavable linker became evident at later timepoints. At 24 h after injection of the compounds, the renal uptake of [^177^Lu]Lu-DOTA-MI-exendin-4 was 3-fold lower than that of the peptide without the cleavable linker. Overall, the uptake in the pancreas and tumor was lower for the cleavable ^177^Lu-labeled peptide, which contrasts with the cleavable peptide labeled with ^68^Ga. The main reason for the different retention profiles is most probably related to the structure of the compounds. The chelator–radionuclide complex may alter the overall charge and structure of the peptide, which may influence renal reabsorption by affecting the peptides’ recognition and binding to endocytic receptors involved in the reabsorption process. Furthermore, conformational changes may also influence the recognition of the cleavable linker by brush border enzymes. Importantly, the reduced uptake of our new compounds in the pancreas and kidney, and the preserved uptake in tumor tissue, improves the ratio between target and non-target organs and could therefore be important in the next step toward PRRT.

Stability studies in human serum revealed slight decomposition of [^68^Ga]Ga-NODAGA-MI-exendin-4 (Figure S1) and [^177^Lu]Lu-DOTA-exendin-4 (Figure S2). One of the metabolites could be an oxidized peptide because exendin-4 has one methionine in its structure and the cleavable linker contains an additional methionine. However, the decomposition profile is not consistent for every peptide, and we did not observe a higher oxidation rate for the peptides with the cleavable linker than for the peptides without. In future studies, oxidation may be prevented by the addition of anti-oxidants, such as ascorbic acid, ethanol, or seleno-methionine, during or after the labeling. Another explanation for the slight decomposition may be the formation of isomers. For the synthesis of the compounds, enantiomerically pure chelators have been used, so we do not expect isomers to be present during or directly after labeling. However, during incubation in human serum at 37 °C, isomers could have been formed as the formation is dependent on pH and temperature.^32^ The formation of isomers may explain the additional peak that is observed just before the peak of the intact radiolabeled compound, which is only observed after the radiolabeled compound is incubated in human serum. In future studies, mass spectrometry could be used to identify the formed metabolites.

In our study, the estimated absorbed dose in mouse and human kidneys for [^68^Ga]Ga-NOTA-MI-exendin-4 was 12% lower than that for [^68^Ga]Ga-NOTA-exendin-4. Although the kidney uptake of [^68^Ga]Ga-NOTA-MI-exendin-4 was 65% less than the uptake of [^68^Ga]Ga-NOTA-exendin-4 4 h p.i., this was not reflected in the estimated absorbed kidney dose values. This might be explained by the short physical half-life of ^68^Ga as the main contributor for the absorbed doses for both peptides. A significant effect on the estimated absorbed dose in the kidneys for the ^177^Lu-labeled compound was observed after introduction of the MI linker (57% lower absorbed dose values as compared to [^177^Lu]Lu-DOTA-exendin). This reduced absorbed kidney dose encourages the use of exendin labeled with beta-emitters.^12^ Next to exendin, several other radiolabeled peptides show predominant renal excretion and retention (e.g., minigastrin or octreotide). Therefore, applying this cleavable linker to other peptides or nanobodies may improve other PRRT approaches as well.^29,33,34^

## Conclusions

[^68^Ga]Ga-NOTA-MI-exendin-4 showed a renal uptake that was 70% lower than [^68^Ga]Ga-NOTA-exendin-4 in BALB/c nude mice, while the radiotracer uptake in the INS-1 tumor was preserved, which could potentially increase the sensitivity of GLP-1R PET/CT to detect insulinomas. Furthermore, the reduced kidney accumulation and absorbed kidney dose of [^177^Lu]Lu-DOTA-MI-exendin-4 might open a new window of opportunity for PRRT using exendin.

## Supporting Information Available

Additional methods of serum stability; additional results of ^68^Ga-labeled NOTA and NODAGA compounds; serum stability graphs for all compounds; peptide characteristics; and biodistribution data (PDF)

## Abbreviations

CT: computed tomography
GLP-1: glucagon-like peptide-1
GLP-1R: glucagon-like peptide-1 receptor
Mal: maleimide
MI: Met-Ile-mal: methionine–isoleucine–N-(2-aminoethyl)maleimide
MVK: methionine–valine–lysine
NEP: neutral endopeptidase
PET: positron emission tomography
PRRT: peptide receptor radionuclide therapy
SPECT: single-photon emission computed tomography
SRS: somatostatin receptor scintigraphy
