# Supplementary material for: New Radiolabeled Exendin Analogues Show Reduced Renal Retention
Source: Mol Pharm. 2023 Jun 2;20(7):3519–28. doi: 10.1021/acs.molpharmaceut.3c00117 (PMC10324399; doi:10.1021/acs.molpharmaceut.3c00117)
Supplement: Supplementary file 1 — mp3c00117_si_001.pdf [file mp3c00117_si_001.pdf]

Supporting information

Materials and methods

Serum stability of ^{68}Ga- and ^{177}Lu-labeled exendin-4 analogues

NOTA-MI-exendin-4, NOTA-exendin-4, NODAGA-MI-exendin-4 and NODAGA-exendin-4 were labeled with ^{68}Ga at a molar activity of 314, 167, 531 and 164 GBq/µmol respectively and quality control and purification were performed as described in the materials and methods of the main text (Section radiolabeling of exendin-4 analogues with ^{68}Ga). DOTA-exendin-4 and DOTA-MI-exendin-4 were labeled with ^{177}Lu at a molar activity of 75 GBq/µmol. The radiolabeled compounds were incubated in human serum (1:10) at 37°C and samples were taken after 1 and 2 hour incubation for the ^{68}Ga-labeled compounds and additional sampling was done after 4 and 24 hours for the ^{177}Lu-labeled compounds. Supernatant was obtained by precipitating the serum proteins by adding a 1:1 (v/v) volume of acetonitrile, followed by centrifugation for 5 min at 3220xg. Subsequently, the supernatant was analyzed using RP-HPLC. RP-HPLC was performed as described in the main text.

Results

Biodistribution of [^{68}Ga]Ga-NOTA-exendin-4, [^{68}Ga]Ga-NOTA-MI-exendin-4, [^{68}Ga]Ga-NODAGA-exendin-4 and [^{68}Ga]Ga-NODAGA-MI-exendin-4 in healthy mice

The complete biodistribution of [^{68}Ga]Ga-NOTA-exendin-4, [^{68}Ga]Ga-NOTA-MI-exendin-4, [^{68}Ga]Ga-NODAGA-exendin-4 and [^{68}Ga]Ga-NODAGA-MI-exendin-4 in healthy C3H mice is presented in Table S3. All compounds show rapid elimination from the circulation. When comparing the kidney uptake of [^{68}Ga]Ga-NODAGA-MI-exendin-4 with [^{68}Ga]Ga-NODAGA-exendin-4, a decreased uptake of almost 58% was found (109 ± 7 %IA/g vs 258 ± 21 %IA/g respectively, $p<0.0001$) four hours after injection. For [^{68}Ga]Ga-NOTA-MI-exendin-4 and [^{68}Ga]Ga-NOTA-exendin-4 this reduction was even higher, 65% (72.7 ± 3.9 %IA/g vs 208 ± 17 %IA/g respectively, $p<0.0001$). In addition, whereas the kidney uptake remained high during four hours for both compounds without the cleavable linker, the uptake

decreased with 39% for [^{68}Ga]Ga-NODAGA-MI-exendin-4 from one to four hours and reduced with even 52% for [^{68}Ga]Ga-NOTA-MI-exendin-4.

Overall, a much lower kidney uptake was observed for both NOTA-conjugated compounds than for the NODAGA-conjugated compounds. Furthermore, a higher pancreas-to-kidney ratio was found for [^{68}Ga]Ga-NOTA-MI-exendin-4 (0.17 ± 0.01) compared to [^{68}Ga]Ga-NODAGA-MI-exendin-4 (0.11 ± 0.01) ($p<0.0001$). Co-injection of unlabeled exendin-4 showed that the uptake of all radiolabeled peptides in the pancreas, lungs and duodenum (transition from stomach to duodenum) could be blocked by an excess unlabeled peptide and therefore was GLP-1 receptor mediated.

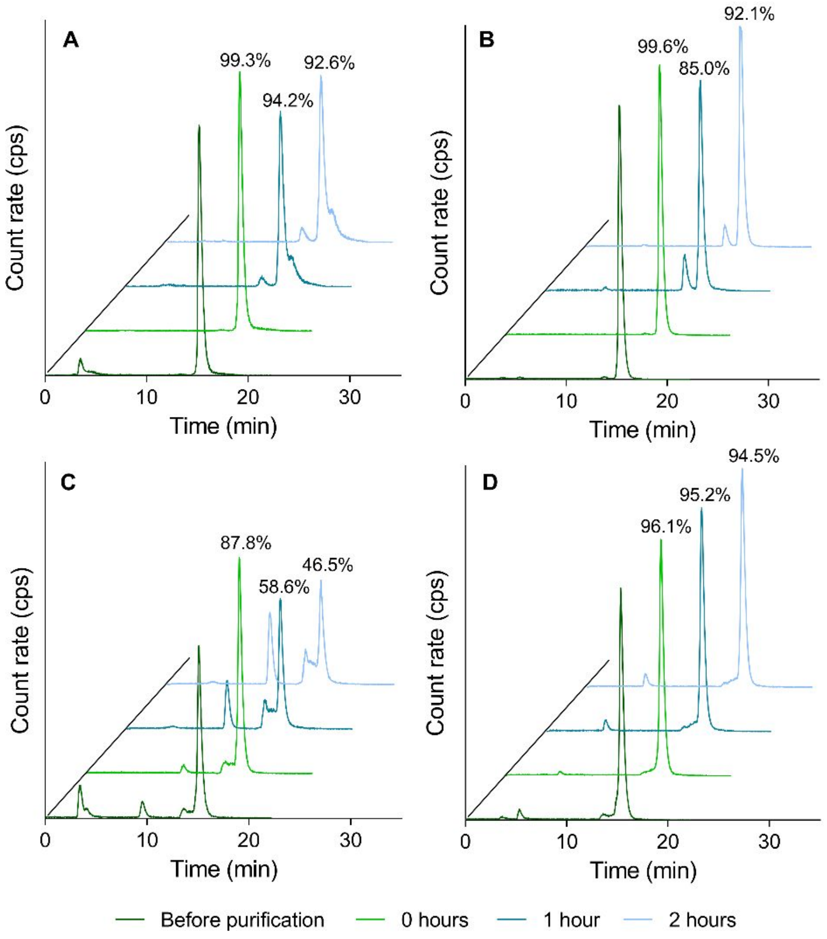

Figure S1. Stability of ^{68}Ga-labeled NODAGA-exendin-4 (**A**), NOTA-exendin-4 (**B**), NODAGA-MI-exendin-4 (**C**) and NOTA-MI-exendin-4 (**D**) before purification, and before and 1 and 2 hours after incubation in human serum at 37°C. Graphs show HPLC profiles.

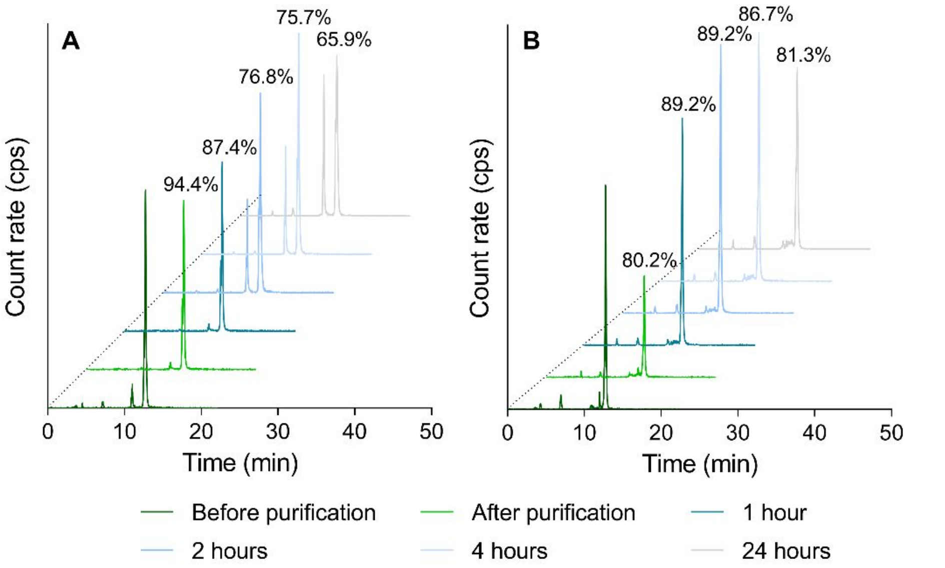

Figure S2. Stability of ^{177}Lu-labeled DOTA-exendin-4 (**A**) and DOTA-MI-exendin-4 (**B**) before purification, and before and 1, 2, 4 and 24 hours after incubation in human serum at 37°C. Graphs show HPLC profiles.

Table S1. Name, abbreviation, molecular mass and amino acid sequence of exendin-4 derivatives.

Peptide	Abbreviation	Molecular weight (Da)	Amino Acid sequence
**[Lys^{40}(DTPA)]-exendin-3**	DTPA-exendin-3	4816.3	HSDGTGTSDLSKQMEEEAVRLFIEWLKNGGPSSGAPPPS-K(DTPA)-NH$_2$
**Exendin-4**	Exendin-4	4186.6	HGEGTFTSDLSKQMEEEAVRLFIEWLKNGGPSSGAPPPS-NH$_2$
**[Lys^{40}(NODAGA)]-exendin-4**	NODAGA-exendin-4	4673.6	HGEGTFTSDLSKQMEEEAVRLFIEWLKNGGPSSGAPPPS-**K(NODAGA-Bn-SCN)**-NH$_2$
**Lys^{40}[Mep((S-)NODAGA-Met-Ile-Mal-)]-exendin-4**	NODAGA-MI-exendin-4	5308.95	HGEGTFTSDLSKQMEEEAVRLFIEWLKNGGPSSGAPPPS-**K(Mep[NODAGA-Bn-SCN-Met-Ile-Mal])**-NH$_2$
**[Lys^{40}(NOTA)]-exendin-4**	NOTA-exendin-4	4765.3	HGEGTFTSDLSKQMEEEAVRLFIEWLKNGGPSSGAPPPS-**K(NOTA-Bn-SCN)**-NH$_2$
**Lys40[Mep((S-)NOTA-Met-Ile-Mal-)]-exendin-4**	NOTA-MI-exendin-4	5237.9	HGEGTFTSDLSKQMEEEAVRLFIEWLKNGGPSSGAPPPS-**K(Mep[NOTA-Bn-SCN-Met-Ile-Mal])**-NH$_2$
**[Lys^{40}(DOTA)]-exendin-4**	DOTA-exendin-4	4866.4	HGEGTFTSDLSKQMEEEAVRLFIEWLKNGGPSSGAPPPS-**K(DOTA-Bn-SCN)**-NH$_2$
**Lys40[Mep((S-DOTA-Met-Ile-Mal-)]-exendin-4**	DOTA-MI-exendin-4	5339	HGEGTFTSDLSKQMEEEAVRLFIEWLKNGGPSSGAPPPS-**K(Mep[DOTA-Bn-SCN-Met-Ile-Mal])**-NH$_2$

Table S2. Biodistribution of ^{68}Ga-labeled NOTA-exendin-4 and NOTA-MI-exendin-4 in BALB/c nude mice bearing a subcutaneous INS-1 tumor. Kidney and tumor data are shown in Figure 4. Values are expressed as percentage injected activity per gram tissue (%IA/g).

	**[^{68}Ga]Ga-NOTA-exendin-4**				**[^{68}Ga]Ga-NOTA-MI-exendin-4**			
	1h (n=5)	2h (n=5)	4h (n=5)	+ excess 4h (n=2)	1h (n=5)	2h (n=5)	4h (n=5)	+ excess 4h (n=2)
**Blood**	0.25 ± 0.04	0.14 ± 0.01	0.11 ± 0.01	0.06 ± 0.01	0.33 ± 0.01	0.22 ± 0.02	0.14 ± 0.01	0.12 ± 0.03
**Muscle**	0.05 ± 0.01	0.04 ± 0.01	0.08 ± 0.05	0.03 ± 0.02	0.04 ± 0.01	0.04 ± 0.02	0.02 ± 0.01	0.03 ± 0.00
**Tumor**	27.8 ± 4.9	27.4 ± 4.7	24.9 ± 9.3	1.45 ± 0.02	29.3 ± 6.9	30.1 ± 9.9	25.0 ± 8.0	1.35 ± 0.25
**Heart**	0.20 ± 0.02	0.15 ± 0.01	0.14 ± 0.02	0.05 ± 0.00	0.24 ± 0.03	0.18 ± 0.02	0.13 ± 0.01	0.08 ± 0.02
**Lung**	4.34 ± 0.85	4.49 ± 0.85	3.14 ± 0.46	0.22 ± 0.04	6.33 ± 1.54	5.77 ± 1.18	3.69 ± 0.64	0.31 ± 0.01
**Spleen**	0.28 ± 0.03	0.30 ± 0.04	0.31 ± 0.07	0.17 ± 0.00	0.28 ± 0.08	0.23 ± 0.04	0.21 ± 0.05	0.08 ± 0.01
**Pancreas**	13.1 ± 2.2	12.3 ± 2.9	10.7 ± 0.9	0.29 ± 0.01	11.4 ± 2.7	10.4 ± 1.9	7.61 ± 2.14	0.30 ± 0.02
**Kidney**	127 ± 17	114 ± 12	128 ± 9	161 ± 3	103 ± 15	73.4 ± 6.7	34.2 ± 4.2	42.5 ± 7.3
**Liver**	0.56 ± 0.03	0.45 ± 0.04	0.53 ± 0.06	0.47 ± 0.06	0.51 ± 0.04	0.43 ± 0.06	0.33 ± 0.04	0.22 ± 0.03
**Stomach**	2.46 ± 0.45	2.03 ± 0.30	2.07 ± 0.27	0.12 ± 0.01	2.86 ± 0.32	2.35 ± 0.14	1.59 ± 0.66	0.20 ± 0.08
**Duodenum**	3.39 ± 1.37	4.00 ± 0.45	4.18 ± 0.59	0.35 ± 0.14	3.89 ± 1.59	4.49 ± 1.15	2.42 ± 1.56	0.35 ± 0.03

Table S3A. Biodistribution of ^{68}Ga-labeled NOTA-exendin-4 and NOTA-MI-exendin-4 in C3H mice. Kidney and pancreas data are shown in Figure 3. Values are expressed as percentage injected activity per gram tissue (%IA/g).

	**[^{68}Ga]Ga-NOTA-exendin-4**				**[^{68}Ga]Ga-NOTA-MI-exendin-4**			
	1h (n=5)	2h (n=5)	4h (n=4)	+ excess 4h (n=2)	1h (n=5)	2h (n=5)	4h (n=5)	+ excess 4h (n=2)
**Blood**	0.36 ± 0.02	0.16 ± 0.01	0.11 ± 0.01	0.10 ± 0.00	0.48 ± 0.05	0.28 ± 0.02	0.17 ± 0.02	0.12 ± 0.00
**Muscle**	0.07 ± 0.00	0.04 ± 0.01	0.05 ± 0.02	0.03 ± 0.01	0.08 ± 0.01	0.05 ± 0.01	0.04 ± 0.02	0.02 ± 0.00
**Heart**	0.29 ± 0.02	0.19 ± 0.02	0.16 ± 0.04	0.09 ± 0.00	0.37 ± 0.04	0.24 ± 0.04	0.15 ± 0.03	0.07 ± 0.01
**Lung**	18.7 ± 1.6	14.6 ± 1.5	12.9 ± 3.1	0.44 ± 0.10	21.0 ± 3.3	19.6 ± 1.3	13.1 ± 1.0	0.45 ± 0.10
**Spleen**	0.27 ± 0.05	0.22 ± 0.02	0.27 ± 0.13	0.21 ± 0.01	0.26 ± 0.03	0.25 ± 0.08	0.15 ± 0.03	0.10 ± 0.01
**Pancreas**	21.5 ± 1.2	20.6 ± 2.3	18.3 ± 3.4	0.36 ± 0.03	22.2 ± 2.3	18.5 ± 0.9	11.6 ± 1.4	0.35 ± 0.05
**Kidney**	203 ± 15	203 ± 11	208 ± 17	250 ± 8	152 ± 17	106 ± 5	72.7 ± 3.9	37.7 ± 12.0
**Liver**	0.43 ± 0.05	0.39 ± 0.02	0.42 ± 0.06	0.50 ± 0.00	0.53 ± 0.06	0.47 ± 0.04	0.37 ± 0.04	0.37 ± 0.14

**Stomach**	5.38 ± 0.43	4.44 ± 0.71	4.58 ± 0.18	0.21 ± 0.01	5.83 ± 0.99	4.53 ± 0.78	3.14 ± 0.10	0.19 ± 0.04
**Duodenum**	5.43 ± 1.25	4.87 ± 1.50	4.52 ± 0.38	0.26 ± 0.02	5.97 ± 0.39	4.79 ± 0.95	3.11 ± 0.58	1.09 ± 0.21

Table S3B. Biodistribution of ^{68}Ga-labeled NODAGA-exendin-4 and NODAGA-MI-exendin-4 in C3H mice. Values are expressed as percentage injected activity per gram tissue (%IA/g).

	**[^{68}Ga]Ga-NODAGA-exendin-4**				**[^{68}Ga]Ga-NODAGA-MI-exendin-4**			
	1h (n=5)	2h (n=5)	4h (n=5)	+ excess 4h (n=2)	1h (n=5)	2h (n=5)	4h (n=5)	+ excess 4h (n=2)
**Blood**	0.37 ± 0.03	0.17 ± 0.01	0.10 ± 0.01	0.05 ± 0.00	0.56 ± 0.06	0.29 ± 0.02	0.21 ± 0.01	0.18 ± 0.01
**Muscle**	0.09 ± 0.03	0.05 ± 0.01	0.05 ± 0.00	0.04 ± 0.01	0.09 ± 0.01	0.05 ± 0.01	0.04 ± 0.01	0.02 ± 0.00
**Heart**	0.29 ± 0.04	0.22 ± 0.06	0.17 ± 0.02	0.06 ± 0.01	0.34 ± 0.04	0.21 ± 0.01	0.15 ± 0.01	0.08 ± 0.01
**Lung**	25.8 ± 3.0	18.6 ± 3.2	12.7 ± 1.5	0.5 ± 0.1	17.9 ± 3.2	11.4 ± 2.0	11.1 ± 2.1	0.65 ± 0.12
**Spleen**	0.27 ± 0.06	0.21 ± 0.01	0.21 ± 0.03	0.12 ± 0.00	0.27 ± 0.02	0.20 ± 0.03	0.18 ± 0.03	0.13 ± 0.01
**Pancreas**	24.3 ± 3.3	22.6 ± 1.9	21.7 ± 2.3	0.43 ± 0.05	16.9 ± 0.7	14.4 ± 1.3	10.2 ± 0.8	0.44 ± 0.02
**Kidney**	256 ± 10	242 ± 15	258 ± 21	276 ± 15	179 ± 8	131 ± 19	109 ± 7	98 ± 8
**Liver**	0.41 ± 0.03	0.37 ± 0.02	0.39 ± 0.05	0.28 ± 0.00	0.74 ± 0.05	0.57 ± 0.03	0.50 ± 0.02	0.39 ± 0.04
**Stomach**	6.05 ± 0.80	5.23 ± 1.01	5.71 ± 1.34	0.22 ± 0.04	4.11 ± 0.63	3.36 ± 0.77	2.67 ± 0.25	0.24 ± 0.01
**Duodenum**	5.98 ± 1.78	5.67 ± 0.84	5.07 ± 1.05	0.22 ± 0.01	4.77 ± 0.96	3.49 ± 1.35	2.38 ± 0.18	0.68 ± 0.26

Table S4A. Biodistribution of [^{177}Lu]Lu-DOTA-exendin-4 in BALB/c nude mice bearing a subcutaneous INS-1 tumor. Values are expressed as percentage injected activity per gram tissue (%IA/g).

	**[^{177}Lu]Lu-DOTA-exendin-4**						
	15min (n=5)	30 min (n=5)	4h (n=5)	24h (n=5)	48h (n=5)	72h (n=5)	+ excess 4h (n=2)
**Blood**	2.53 ± 0.84	0.87 ± 0.19	0.10 ± 0.01	0.03 ± 0.00	0.01 ± 0.00	0.01 ± 0.00	0.10 ± 0.01
**Muscle**	0.56 ± 0.10	0.19 ± 0.07	0.03 ± 0.01	0.02 ± 0.00	0.02 ± 0.00	0.01 ± 0.00	0.02 ± 0.00
**Tumor**	23.4 ± 5.9	23.7 ± 6.4	16.5 ± 5.9	11.3 ± 2.0	10.8 ± 4.5	6.86 ± 2.86	1.32 ± 0.23
**Heart**	0.98 ± 0.17	0.41 ± 0.16	0.48 ± 0.88	0.06 ± 0.01	0.05 ± 0.01	0.04 ± 0.00	0.07 ± 0.00
**Lung**	9.11 ± 2.21	8.47 ± 4.82	2.91 ± 1.67	1.91 ± 0.28	1.29 ± 0.30	1.02 ± 0.24	0.25 ± 0.01
**Spleen**	0.70 ± 0.32	0.32 ± 0.05	0.16 ± 0.03	0.14 ± 0.02	0.14 ± 0.04	0.10 ± 0.01	0.12 ± 0.00
**Pancreas**	11.8 ± 2.4	13.7 ± 2.5	12.5 ± 2.8	8.16 ± 0.36	5.38 ± 0.45	4.71 ± 0.99	0.30 ± 0.05
**Kidney**	89.8 ± 9.0	119 ± 19	125 ± 17	99.3 ± 9.2	58.6 ± 4.3	45.8 ± 3.9	143 ± 5
**Liver**	1.07 ± 0.35	0.56 ± 0.10	0.30 ± 0.07	0.20 ± 0.03	0.14 ± 0.01	0.14 ± 0.02	0.25 ± 0.03
**Stomach**	3.06 ± 0.71	2.72 ± 0.96	1.80 ± 0.33	1.39 ± 0.41	0.99 ± 0.06	0.91 ± 0.19	0.10 ± 0.01
**Duodenum**	6.16 ± 2.17	5.62 ± 1.97	4.26 ± 1.27	4.04 ± 0.97	3.13 ± 0.90	3.48 ± 1.04	0.31 ± 0.04
**Colon**	1.35 ± 0.43	0.84 ± 0.16	0.53 ± 0.15	0.30 ± 0.06	0.21 ± 0.04	0.17 ± 0.03	0.03 ± 0.00
**Femur**	0.40 ± 0.12	0.16 ± 0.03	0.05 ± 0.02	0.02 ± 0.01	0.03 ± 0.01	0.01 ± 0.01	0.03 ± 0.00
**Bone marrow**	0.57 ± 0.39	0.25 ± 0.15	0.14 ± 0.05	0.11 ± 0.02	0.13 ± 0.04	0.12 ± 0.03	0.14 ± 0.01

Table S4B. Biodistribution of [^{177}Lu]Lu-DOTA-MI-exendin-4 in BALB/c nude mice bearing a subcutaneous INS-1 tumor. Values are expressed as percentage injected activity per gram tissue (%IA/g).

	**[^{177}Lu]Lu-DOTA-MI-exendin-4**						
	15min (n=5)	30 min (n=5)	4h (n=5)	24h (n=5)	48h (n=5)	72h (n=5)	+ excess 4h (n=2)
**Blood**	2.01 ± 0.20	1.05 ± 0.12	0.17 ± 0.02	0.03 ± 0.00	0.01 ± 0.00	0.01 ± 0.00	0.13 ± 0.01
**Muscle**	0.37 ± 0.05	0.14 ± 0.03	0.02 ± 0.00	0.01 ± 0.00	0.01 ± 0.00	0.00 ± 0.00	0.02 ± 0.00
**Tumor**	15.9 ± 2.4	18.9 ± 4.2	13.4 ± 2.5	7.81 ± 1.54	4.24 ± 0.63	3.65 ± 1.74	0.61 ± 0.06
**Heart**	0.78 ± 0.11	0.40 ± 0.01	0.12 ± 0.02	0.04 ± 0.01	0.03 ± 0.00	0.01 ± 0.00	0.08 ± 0.00
**Lung**	7.59 ± 2.34	5.34 ± 0.94	1.99 ± 0.59	1.26 ± 0.43	0.78 ± 0.27	0.35 ± 0.17	0.34 ± 0.01
**Spleen**	0.52 ± 0.10	0.37 ± 0.19	0.15 ± 0.04	0.07 ± 0.01	0.05 ± 0.00	0.04 ± 0.01	0.13 ± 0.01
**Pancreas**	8.63 ± 1.34	8.29 ± 1.03	6.35 ± 1.20	1.79 ± 0.32	1.03 ± 0.10	0.58 ± 0.06	0.28 ± 0.03
**Kidney**	90.5 ± 6.2	106 ± 7	103 ± 12	39.4 ± 6.3	22.6 ± 3.3	13.0 ± 2.5	100 ± 10
**Liver**	0.99 ± 0.12	0.77 ± 0.41	0.24 ± 0.03	0.12 ± 0.02	0.09 ± 0.01	0.07 ± 0.01	0.35 ± 0.00
**Stomach**	2.70 ± 0.86	1.87 ± 0.13	1.00 ± 0.05	0.55 ± 0.17	0.38 ± 0.04	0.20 ± 0.01	0.11 ± 0.00
**Duodenum**	4.26 ± 1.11	4.41 ± 1.52	2.42 ± 1.13	1.24 ± 0.15	1.00 ± 0.44	0.75 ± 0.23	0.32 ± 0.06
**Colon**	0.79 ± 0.26	0.60 ± 0.05	0.31 ± 0.05	0.16 ± 0.02	0.12 ± 0.02	0.06 ± 0.01	0.09 ± 0.00
**Femur**	0.35 ± 0.09	0.16 ± 0.03	0.04 ± 0.01	0.04 ± 0.05	0.01 ± 0.00	0.01 ± 0.01	0.05 ± 0.00
**Bone marrow**	0.54 ± 0.11	0.34 ± 0.08	0.14 ± 0.03	0.09 ± 0.04	0.07 ± 0.03	0.04 ± 0.02	0.28 ± 0.09
